# A Submicron-Scale Plugging Agent for Oil-Based Drilling Fluid Synthesized Using the Inverse Emulsion Polymerization Method

**DOI:** 10.3390/polym15132815

**Published:** 2023-06-26

**Authors:** Zhiquan Zhang, Baimei Dai, Peng Xu

**Affiliations:** School of Petroleum Engineering, Yangtze University, Wuhan 430100, China

**Keywords:** polymer microspheres, nano-scale, oil-based drilling fluid, plugging agent, inverse emulsion polymerization

## Abstract

Due to the increasing difficulty of drilling in the later stages of oil and gas field development, the development of micro-pores and micro-fractures is becoming common. Conventional plugging agents have relatively large particle sizes. So, choosing the appropriate plugging agent can prevent leakages. Using the inverse emulsion polymerization method, acrylamide, 2-acrylamide-2-methylpropane sulfonic acid and acrylic acid were selected to be the main reaction monomers, N,N′-methylenebisacrylamide was used as a crosslinking agent, sorbitan monostearate and polyoxyethylene sorbitan anhydride monostearate were used as emulsifiers, and 2,2′-azobis(2-methylpropionamidine) dihydrochloride was used as the initiator to synthesize a nano-scale plugging agent for oil-based drilling fluid. The plugging agent was characterized using infrared spectroscopy, scanning electron microscopy, and thermogravimetry analysis. The results showed that the plugging agent is spherical and uniform in size, with particles being in the submicron range. Additionally, it exhibited strong temperature resistance. Finally, the performance of the plugging agent was evaluated via experiments conducted under normal temperature and pressure, high-temperature and high-pressure, and core-plugging conditions. After adding the plugging agent to the oil-based drilling fluid, the basic rheological properties of the oil-based drilling fluid were not significantly affected. Furthermore, the filtration loss was significantly reduced under normal temperature and pressure, as well as under high-temperature and high-pressure conditions, after aging. When the plugging agent with 3% concentration was added, the reduction rate of pore core permeability reached 96.04%. Therefore, the plugging agent for the oil-based drilling fluid can effectively improve the wellbore stability and has a promising potential for field applications.

## 1. Introduction

Currently, as the extraction of oil and gas gradually shifts towards deeper water and unconventional oil and gas reservoirs, well leakages remains one of the most complex challenges in the drilling process [1,2,3] (Cui and fluld, 2005; Lin and Wang, 2014; Z. J. S.-G. E. Wang, 2012). Borehole instability is prone to occur in porous or fractured formations. Strengthening the plugging capacity of drilling fluid is one of the methods to enhance borehole stability. Severe wellbore instability can hinder drilling operations and pose a safety risk. The permeability of deep formations is low, and there are widespread nanoscale micro-cracks. The drilling fluid will enter the deep stratum along the cracks and cause wellbore instability and the production of waste drilling fluid and increase the difficulty of construction, and these sites more prone to stuck drilling accidents [4]. Compared to water-based drilling fluid, oil-based drilling fluid has a higher cost and limited environmental adaptability. However, it exhibits good thermal stability, which is beneficial for maintaining borehole wall stability and causing less damage to oil and gas formations. In challenging scenarios, such as high-temperature deep wells, highly inclined directional wells, horizontal wells, and complex formations, the advantages of oil-based drilling fluid make it a crucial choice for effective protection [5,6,7].

To solve the problem of oil-based drilling fluid leakages, using plugging materials while drilling is generally adopted to realize plugging according to bridging theories [8,9,10]. Up to now, the main plugging agents used for oil-based drilling fluid include rigid plugging agents and bituminous flexible plugging agents [11]. However, rigid plugging agents have poor deformability, and bituminous flexible plugging agents have limited pressure resistance. As a result, when they are used in the field, they struggle to effectively adapt to formations with pores and micro-fractures, leading to a low success rate during plugging. For deep oil and gas reservoir development, the size of the plugging material is very important [12,13,14]. Since 2016, numerous major oil service companies have been actively researching smart gel plugging technologies, utilizing materials that do not require good gradability and are suitable for plug-and-drill applications. Polymer microspheres, a deformable smart gel plugging material, were initially employed in the field as displacement agents to enhance oil recovery. Subsequently, they were utilized as plugging agents and fluid loss reducers in water-based drilling fluid [15,16,17,18].

Nowadays, widely used leak plugging materials in oil fields are primarily acrylamide polymers [19,20]. In recent years, efforts have been made to introduce certain monomers to inhibit the hydrolysis of the amide group within the molecule. This aims to enhance the salt resistance and temperature resistance of the acrylamide polymer, as well as to facilitate better gradability when it enters micro-fractures. Song et al. successfully prepared acrylamide/2-acrylamide-2-methylpropane sulfonic acid/N-Vinyl-2-pyrrolidone(AM/AMPS/NVP) for the ternary copolymerization of a temperature-resistant filtrate loss reducer using free radical aqueous solution polymerization. The research showed that P(AM/AMPS/NVP) had better temperature resistance and filtrate loss performances than P(AM/AMPS) did [21]. Liu et al. used acrylamide and 2-acrylamide-2-methylpropane sulfonic acid as monomer raw materials and synthesized a spherical gel with a temperature resistance of 80 °C, which could not meet the temperature resistance performance with the increase in depth [22]. Zhong et al. synthesized polymer microspheres SPM via suspension polymerization. The plugging agent could absorb oil and expand, but the addition of SPM had a significant impact on the viscosity of the oil-based drilling fluid [23]. Using acrylamide as the main synthetic monomer, Xiang et al. synthesized a gel microsphere emulsion (OBMG) using the inverse emulsion polymerization method. When the effective gel microsphere addition is 2~3%, the plugging effect of oil-based drilling fluid is the best, and it can effectively plug micron cracks [24]. Liu et al. used suspension polymerization to synthesize micron-sized amphiphilic polymer gel microsphere suspensions, which were superior to bitumen oxide and fine-mesh calcium carbonate in terms of rheology for oil-based drilling fluid [25]. Li et al. synthesized gel microspheres for water-based drilling fluid using the inverse emulsion polymerization method. The size was 4.5~68 μm, and the initial thermal decomposition temperature was 150 °C. A temperature rise would increase the filtration capacity of a water-based slurry with gel microspheres [26]. Gao et al. synthesized β-cyclodextrin polymer microspheres with an average particle size of 42.88 μm using β-cyclodextrin as a raw material and Epichlorohydrin as a crosslinker via inverse emulsion polymerization. After hot rolling at 180 °C and 200 °C, the bentonite slurry can effectively reduce the filtration loss, which makes it better than the typical anti-high temperature filtration loss reducer. In addition, β-cyclodextrin polymer microspheres have a minimal effect on the rheological properties of drilling fluid [27]. Xu et al. prepared an internal rigid and external flexible sealer (PANS) with nano-silicon dioxide as a rigid core and poly (acrylamide-Co-N-vinylpyrrolidone) as a flexible shell via reverse-phase emulsion polymerization, which could effectively reduce the filtration losses of bentonite water-based drilling fluids under low-temperature and low-pressure (API) and high-temperature and high-pressure (HTHP) conditions. In addition, the sealing efficiency of 100 °C hot-rolled PANS on 120 mesh sand beds is 4.180% higher than that of pure nano silica (500 nm) [28]. 

**Figure 1:**
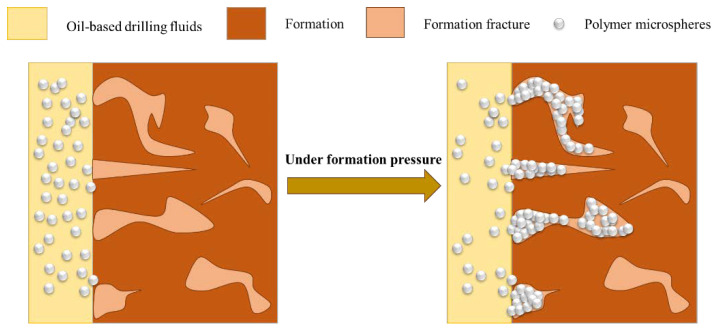
Blocking mechanism of polymer microspheres.

Acrylamide polymer microspheres employed as plugging materials are mainly used in water-based drilling fluids [29,30]. Compared with conventional plugging materials, polymer microspheres are small in size and uniform in particle size [31,32]. During drilling, under the influence of formation pressure difference, acrylamide polymer microspheres can infiltrate the leakage channels, absorb water, and expand. This process leads to the formation of a dense pressure sealing layer in conjunction with other plugging materials. Consequently, the pressure transfer rate is effectively reduced, preventing further invasion of the drilling fluid, improving the success rate of plugging, and ultimately, achieving the objective of stabilizing the wellbore (see Figure 1). In this study, polymer microspheres used in oil-based drilling fluid were synthesized via inverse emulsion polymerization. This was conducted to overcome the compatibility issues between hydrophilic polymer microspheres and the oil-based drilling fluid. The excellent elastic deformation capacity of the synthesized polymer microspheres was effectively utilized to address the challenge of submicron porosity gradation.

## 2. Materials and Methods

### 2.1. Materials Selection

Experimental drugs: Acrylamide (AM), 2-Acrylamido-2-methylpropane sulfonic acid (AMPS), acrylic acid (AA), N,N′-methylenebisacrylamide (MBA), 2,2′-azobis(2-methylpropionamidine) dihydrochloride (AIBI), sodium hydroxide, anhydrous ethanol, sorbitan monostearate (Span60), and polyoxyethylene sorbitan anhydride monostearate (Tween60), pure analysis grade, China Shanghai Aladdin Biochemical Technology Co., Ltd., Shanghai, China. 3# white oil, industrial grade, China Jingzhou Jiahua Technology Co., LTD., Jingzhou, China.

Experimental instruments: A Magnetic Agitator (HJ-2B) displaying an even-number constant temperature, China Changzhou Aode Instrument Manufacturing Co., Ltd., Changzhou, China. A six-speed rotating viscometer, high-temperature and high-pressure filtration instruments, and magnetic stirrers, China Qingdao Dream Instrument Co., Ltd., Qingdao, China. An electronic constant temperature Stainless Steel Water Bath, China Shanghai Yulong Instrument Equipment Co., Ltd., Shanghai, China. An optical microscope made by China Jiangnan Yongxin Optical Co., Ltd., Jiangnan, China. A Scanning Electron Microscope (SU8010), Hitachi, Tokyo, Japan.

### 2.2. Monomer Selection

The main synthetic monomers of polymer microspheres are acrylamide (AM), 2-acrylamide-2-methylpropyl sulfonic acid (AMPS), acrylic acid (AA), and N,N′-methylenebisacrylamide (MBA).

**Figure 2:**
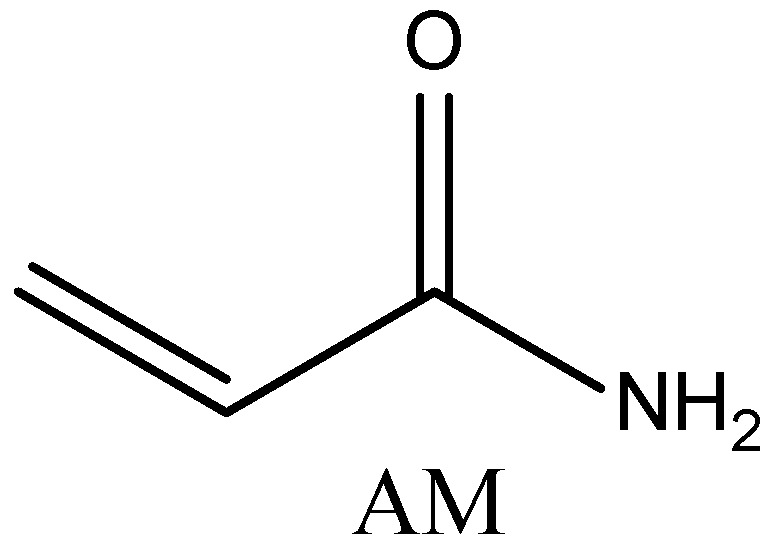
The structural formula of AM.

**Figure 3:**
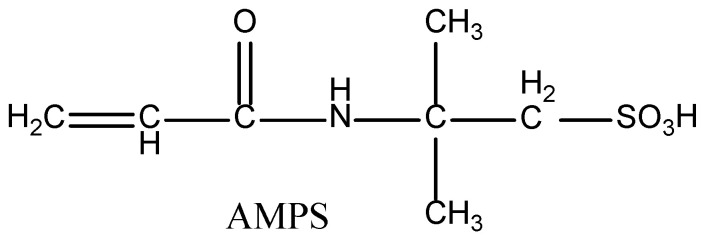
The structural formula of AMPS.

**Figure 4:**
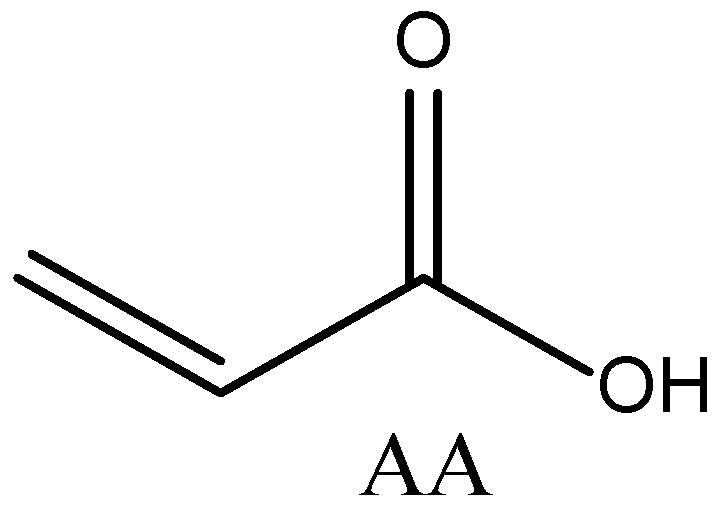
The structural formula of AA.

**Figure 5:**
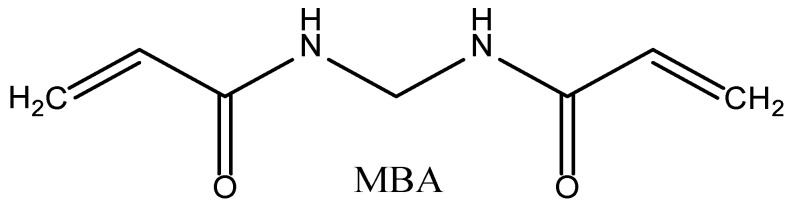
The structural formula of MBA.

(1) The chemical properties of acrylamide (AM) are active, the structure of the carbon–carbon double bond can produce a polymerization reaction with most vinyl monomers, and its branch chain contains only one amide functional group; the synthesis reaction will reflect strong adsorption (as shown in the Figure 2).

(2) 2-Acrylamido-2-methylpropane sulfonic acid (AMPS) contains sulfonic acid groups, and easy copolymerization can ensure the thermal stability of the polymer. The side chain, (CH)₂CH3SO₃, also increased the rigidity of the main chain and increased the polymer’s temperature and salinity resistance (as can be seen in Figure 3).

(3) Acrylic acid (AA) is an unsaturated carboxylic acid that dissolves in water and polymerizes with itself or other vinyl monomers. Its carboxylic acid group, as a hydration group, has a strong adsorption ability (see Figure 4).

(4) N,N′-methylenebisacrylamide (MBA) used as a crosslinking agent, the left and right ends of which each contain a carbon–carbon double bond, in the synthesis process can make the polymer form a three-dimensional network structure, which improves the thermal stability of the polymer (as shown in the Figure 5).

### 2.3. Polymerization Process

**Figure 6:**
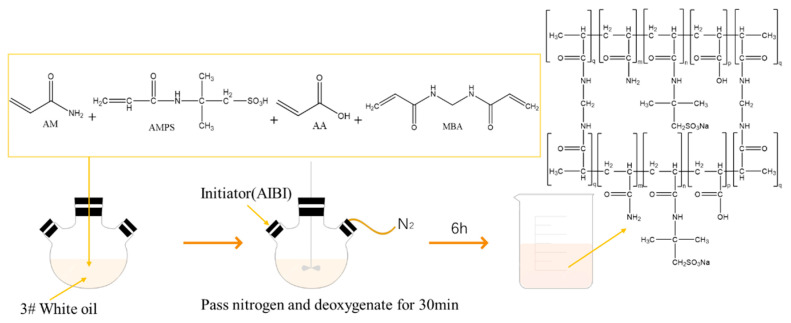
Synthesis of polymer microspheres via inverse emulsion polymerization.

(1) Water phase: Add 100 mL deionized water into 250 mL beaker on a magnetic stirrer, add 30 g AM, 15 g AMPS, and 9 g AA to the beaker, turn on the magnetic stirrer, stir it for 10 min until the monomer is completely dissolved; then, use NaOH to adjust the PH value to neutral. Add 0.13 g crosslinking agent MBA and 0.7 g emulsifier Tween60 to the beaker. The water phase can be obtained by stirring continuously for 20 min until the reaction is complete.

(2) Oil phase: Add 80 mL of 3# white oil into 250 mL beaker, put it on the magnetic stirrer, start stirring, slowly add 6.5 g emulsifier Span60, and keep stirring it for 20 min.

(3) Finally, slowly drop the water phase into the stirring oil phase, keep stirring it at a high speed for 5 min, set the rotational speed to 1000 rpm, and then pour it into a three-way flask; put one end of the flask into nitrogen deoxygenation for about 30 min, insert the agitator into the middle of the flask mouth, and then add 0.09 g initiator AIBI. Finally, turn on the water bath heater, and set the temperature to 55 °C, open the stirrer, and set the speed to 600 rpm. Allow the reaction to proceed for 6 h to obtain emulsion polymer microspheres (see Figure 6).

### 2.4. Mechanism of Polymerization

**Figure 7:**
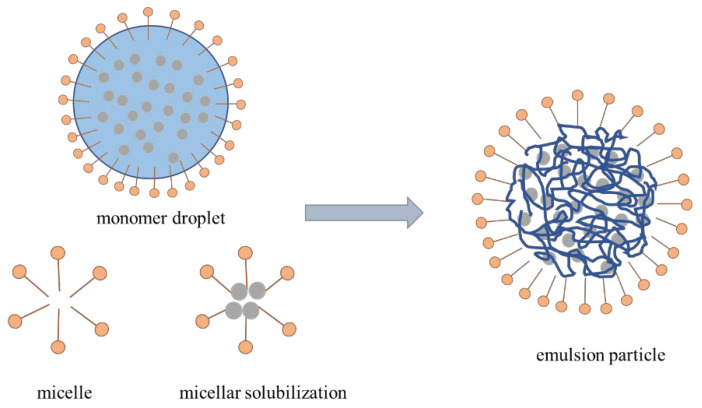
Mechanism of inverse emulsion polymerization.

Inverse emulsion polymerization technology was first proposed by Vanderhoff in 1962. The traditional emulsion consists of an oleophilic monomer as the inner phase and water as the outer phase. The addition of an emulsifier combines the inner phase with the outer phase to form an oil-in-water (O/W) emulsion, in which, without a complete reaction, the structure of monomer droplets and micelles also forms an oil-in-water emulsion. The composition of the continuous phase and dispersed phase of the polymer microsphere is opposite to that of the traditional emulsion, but in terms of the reaction mechanism, both of them are free radical reactions generated by the initiator. The chain free radical enhances growth via the addition of the new monomer free radical and forms the polymer chain. At this time, the chain transfer is accompanied by the formation of branch chains, and the relative molecular weight decreases (as shown in Figure 7) [33]. There are four main stages in the polymerization process: (1) The dispersion stage. In the emulsifying system, the emulsifier dissolves in water and takes the form of a single molecule. After the critical micellar concentration is exceeded, the amount of emulsifier continues to increase, and the micellar form appears in the solution. (2) The latex particle formation stage. The number of monomer droplets in the system is small, and the number of monomer bead drops in the system is low, and the number of micelles is high; so, free radicals decompose and enter the micelles during the reaction. The free radicals initiate a reaction in the micelles to form polymer chains, causing the micelles to swell into emulsion colloidal particles, also known as reaction site latex particles. (3) The latex particle growth stage. In this stage, the initiator continues to release free radicals, the number of latex particles is much higher than that of monomer beads; first, free radicals are consumed in the latex particle consumption monomer reaction, the latex particle volume increases, the number of monomers reduces, and monomer beads in the monomer are also consumed or gradually disappear. (4) The polymerization completion stage. Then it is entering the polymerization completion stage, the system no longer contains micelles and monomer droplets. When the polymer concentration continues to increase and the reaction temperature is reached, the internal polymer chain and the remaining monomer no longer react, and the reaction process is terminated.

### 2.5. Methods Selection

(1) Infrared characterization of polymer microspheres. We took 20 mL of the synthesized emulsion and slowly poured it into 100 mL of anhydrous ethanol for stirring to break the emulsion, put it into the centrifuge, set the speed to 10,000 rpm/min for 20 min, and finally, took the bottom layer precipitation and dried it and crushed it to obtain the final sample, and then, according to the Chinese standard, GB/T 6040-2002, “infrared spectrum analysis methods of general rules Part 5: sample preparation method”, we employed the potassium bromide tablet method using a Fourier transform infrared spectrometer to characterize the analysis.

(2) The microsphere morphology was observed under a scanning electron microscope. The quantitative polymer microsphere emulsion was demulsified, washed, and fully dried, and then microspheres powder was taken for microscopic analysis using a scanning electron microscope (SEM) according to Chinese standard, GB/T 36422-2018, Determination of Micromorphology and diameter.

(3) Thermogravimetric analysis of the thermal stability of microspheres. The thermal stability of 5 mg microsphere powder was studied using a differential thermogravimetric synchronous analyzer at a set temperature of 0~600 °C, protected by inert gas, and the heating rate was 10 °C/min. According to the Chinese standard, “NB/SH/T 0859-2013 Thermal analysis for the determination of thermal stability of chemical substances”, the TG curve and corresponding data obtained from the experiment were analyzed.

(4) High-temperature and high-pressure filtration measurement. After the drilling fluid was configured according to the corresponding formula, it was hot rolled in the rolling furnace for 120 °C × 16 h, and then the tightness of the high-temperature and high-pressure instrument was checked. The aging drilling fluid was poured into the mud cup and assembled according to the requirements. The temperature was set at 120 °C, the upper pressure was set at 4.2 MPa, and the lower pressure was set at 0.7 MPa. After the reading had been taken, the stem was closed and the pressure was relieved, and the instrument was finally disassembled.

(5) Core permeability measurement. The plugging performance of polymer microspheres was evaluated using a liquid measuring device. The pore core of 2.5 × 2.5 cm was put into the core holder, the advection pump was opened, and the confining pressure was 3 MPa to measure its permeability. Then, the oil-based drilling fluid that has been added to the polymer microspheres was poured into the intermediate container, the confining pressure remained unchanged, and nitrogen was injected into one end for water flooding to measure the permeability change.

(6) Rheological performance test. The constant speed device and variable speed device of a rotational viscometer are collectively referred to as the rotating section. An outer cylinder was fixed on the rotating component, which is known as the outer cylinder rotation. The measuring device consists of a measuring spring component, a dial, and an inner cylinder. When the rheological properties of drilling fluid are measured, the viscosity of the oil-based drilling fluid causes the inner cylinder connected to the torsion spring to rotate by a certain angle. According to Newton’s law of internal friction, the magnitude of the rotation angle is directly proportional to the viscosity of the drilling fluid. Therefore, the measurement of oil-based drilling fluid viscosity is transformed into the measurement of the rotation angle of the inner cylinder. The magnitude of the rotation angle can be directly read from the dial. The six-speed rotational viscometer was manufactured according to the specifications of the American Petroleum Institute, which can measure various rheological parameters, and its variable speed range is 3, 6, 100, 200, 300, and 600 rpm/min. The shear rates corresponding to the six rotational speeds of this viscometer are as follows:

3 rpm/min corresponds to a shear rate of 5.11 s^−1^.

6 rpm/min corresponds to a shear rate of 10.22 s^−1^.

100 rpm/min corresponds to a shear rate of 170.3 s^−1^.

200 rpm/min corresponds to a shear rate of 340.7 s^−1^.

300 rpm/min corresponds to a shear rate of 511 s^−1^.

600 rpm/min corresponds to a shear rate of 1022 s^−1^.

The calculation of the apparent viscosity (AV), plastic viscosity (PV), and yield point (YP) of the oil-based drilling fluid based on the dial readings was primarily achieved using the following formulas:

(1) AV(mPa·s)=Φ600/2

(2) $$PV\left(mPa\cdot s\right)=\Phi 600-\Phi 300$$

(3) $$YP\left(Pa\right)=\frac{\Phi 300-PV}{2}$$

where *Φ*600 and *Φ*300 are the readings of the six-speed rotational viscometer at 600 and 300 rpm/min, respectively. The testing of the oil-based drilling fluid needs to be conducted at a consistent temperature of approximately 60 °C throughout the process.

## 3. Results

### 3.1. Scanning Electron Microscope of Polymer Microspheres

A small quantity of polymer microspheres powder was applied onto conductive paper and sprayed with gold. Subsequently, the samples were placed in the sample room, and measurement parameters were configured. The microscopic morphology of the samples was observed, and the size range was measured.

**Figure 8:**
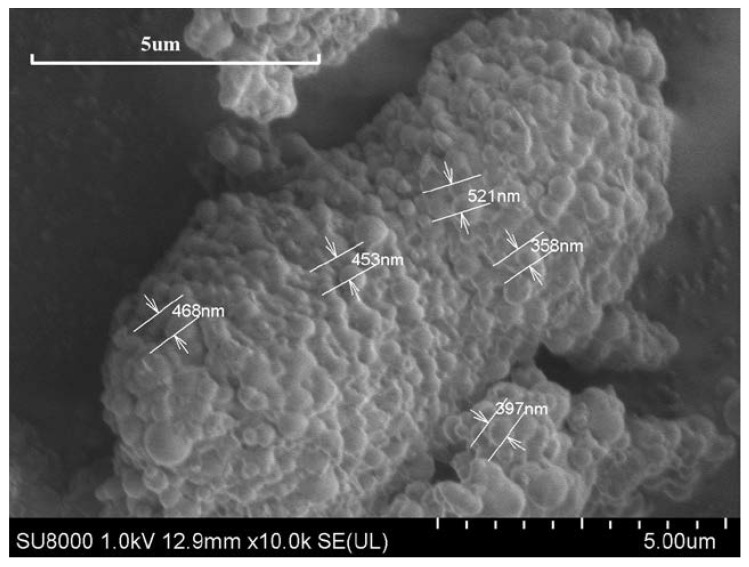
Scanning electron microscope of polymer microspheres.

As can be seen in Figure 8, the polymer microspheres prepared via inverse emulsion polymerization in the experiment were spherical, with uniform particle sizes ranging from 300 nm to 600 nm on average. Therefore, during the drilling process, polymer microspheres, in comparison to those of traditional plugging materials, exhibit a superior capability to penetrate submicron-level pores and fractures within the formation. Additionally, they possess good gradability. When they are subjected to pressure, a dense plugging layer is formed, which improves the success rate of plugging.

### 3.2. Infrared Spectroscopic Analysis of Polymer Microspheres

**Figure 9:**
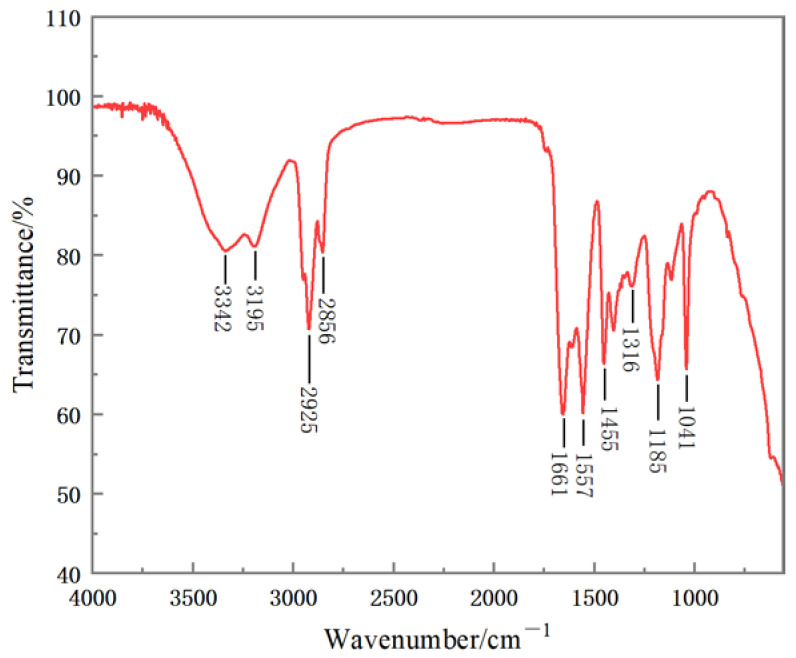
Infrared spectroscopic analysis of polymer microspheres.

According to the infrared spectroscopic analysis of the synthesized product (as shown in the Figure 9), the -NH- medium–strong characteristic absorption peak of AM is located at 3342 cm^−1^. the stretching vibration absorption peak of -NH- in AMPS is at 3195 cm^−1^. At 2925 cm^−1^ and 2856 cm^−1^, there are methylene CH_2_- antisymmetric stretching vibration absorption peaks and symmetric stretching vibration absorption peaks, respectively. At 1661 cm^−1^ and 1557 cm^−1^, there are absorption peaks of -COO- and the characteristic absorption peaks of -COOH, respectively. At 1455 cm^−1^, there is the characteristic absorption peak of AM -CONH_2_. At 1185 cm^−1^, there is the asymmetric stretching vibration absorption peak of the sulfonic acid group in AMPS. The absorption peak of -S-O- in AMPS is at 1041 cm^−1^. The out-of-plane bending vibration absorption peaks of =CH and =CH_2_ did not appear. According to infrared spectrum analysis, it can be concluded that the polymer microspheres synthesized with AM, AMPS, and AA as the main monomers contain the corresponding functional groups designed, which proves that the expected products were obtained in the inverse emulsion polymerization experiment.

### 3.3. Thermogravimetric Analysis of Polymer Microspheres

**Figure 10:**
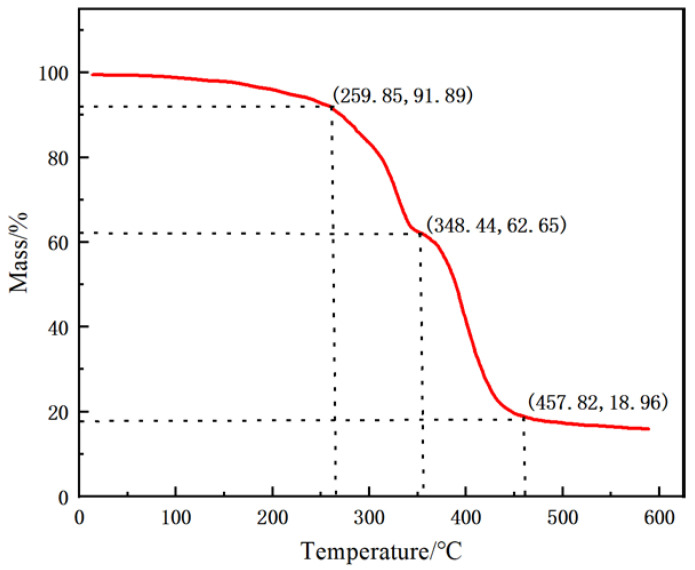
Thermogravimetric analysis of polymer microspheres.

As shown in Figure 10, the weight loss process of thermal decomposition of polymer microspheres can be divided into three stages: (1) In the first stage, when the TG curve is between 0 and 259 °C, the mass of polymer microspheres is reduced by heat, mainly due to the evaporation of water absorbed by sulfonic acid groups and amide groups. (2) In the second stage between 259 °C and 348 °C, the mass of polymer microspheres decreases continuously due to the decomposition of amide groups. (3) In the third stage, between 348 °C and 457 °C, the TG curve decreases because the main chain of the polymer microsphere begins to break and the structure is damaged. Therefore, fewer polymer microspheres are lost in the first stage and have strong temperature resistance.

## 4. Discussion

According to the experimental part, polymer microspheres were synthesized and added to oil-based drilling fluid for performance evaluation. The oil-based drilling fluid formula was as follows: 80/20 oil/water ratio (3# white oil) + 4% main emulsifier + 1% auxiliary emulsifiers + 2% wetting agent + 2.5% calcium oxide + brine (25% mass volume concentration) + 2% organic soil + polymer microspheres in different concentrations.

### 4.1. Effect of Polymer Microspheres on Properties of Oil-Based Drilling Fluid

Different concentrations of polymer microspheres were added to the oil-based drilling fluid. After aging at 120 °C × 16 h, the impact of polymer microspheres on the rheological properties of the oil-based drilling fluid was observed by comparing the changes in apparent viscosity (AV) and plastic viscosity (PV) before and after aging. Subsequently, the effects of adding different concentrations of polymer microspheres on the normal pressure filtration loss (FL_API_) and high-temperature and high-pressure filtration loss (FL_HTHP_) of oil-based drilling fluid were compared to determine the optimal polymer microsphere addition amount.

**Figure 11:**
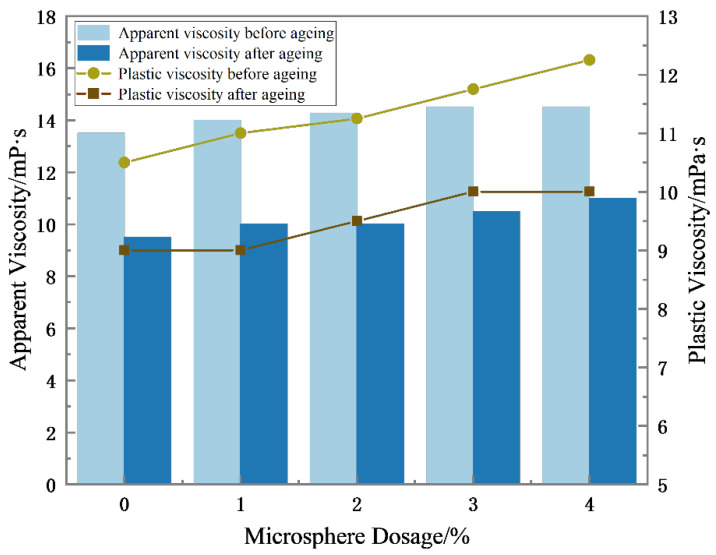
Effect of polymer microspheres concentration on rheological properties of oil-based drilling fluid.

The rheological parameters of drilling fluid serve as crucial indicators for assessing the drilling fluid system and its performance. They play an important role in ensuring drilling safety, quality, and efficiency. As can be seen from Figure 11, with the increase in polymer microsphere concentration, the effective content of polymer microspheres will lead to a decrease in the oil–water ratio of the oil-based drilling fluid. In the figure, the apparent viscosity and plastic viscosity of oil-based drilling fluid both show a slight increase trend, but the variation range is small. It can be seen that the addition of different amounts of polymer microspheres has no significant effect on the rheological properties of oil-based drilling fluids before and after aging at 120 °C × 16 h.

**Figure 12:**
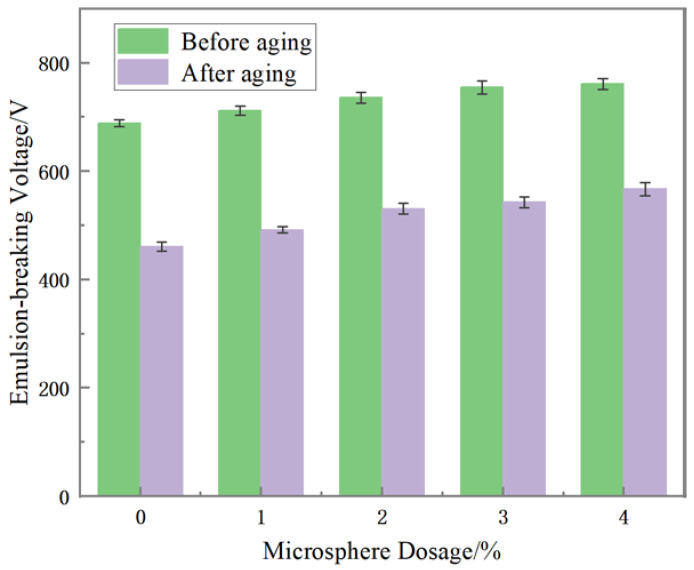
Effect of polymer microspheres concentration on the stability of oil-based drilling fluid.

As can be seen from Figure 12, with the increase in the polymer microspheres concentration, the emulsion-breaking voltage of oil-based drilling fluid tends to rise before and after aging. It can be seen that the addition of polymer microspheres will not hurt the emulsion-breaking voltage of oil-based drilling fluid, but, it improves the emulsion stability. The polymer microspheres emulsion added to the oil-based drilling fluid is the water-in-oil emulsion itself, which exhibits excellent compatibility with oil-based drilling fluid. The submicron particles can disperse effectively at the oil–water interface, thereby enhancing the emulsion stability.

**Figure 13:**
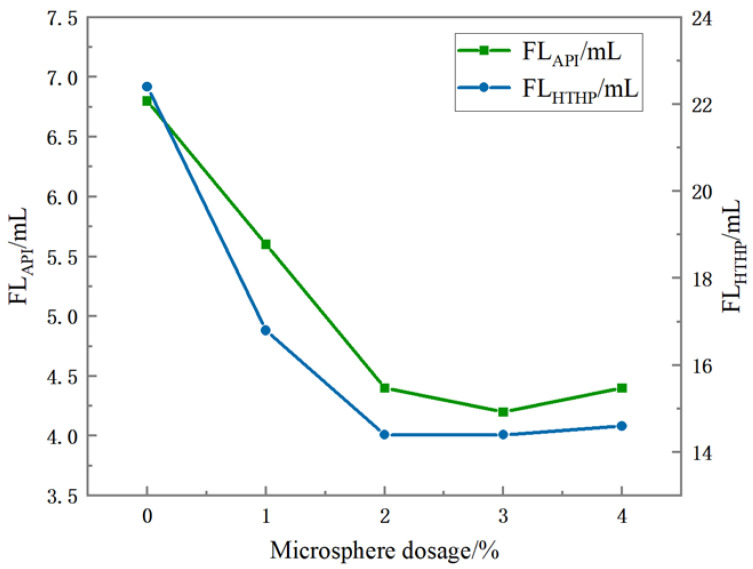
Effect of polymer microspheres concentration on filtration performance of oil-based drilling fluid.

It can be seen from Figure 13 that the addition of polymer microspheres of different concentrations reduces the filtration performance of oil-based drilling fluid to a certain extent. At a normal temperature and pressure, the static filtration loss (API filtration) was reduced from 6.8 mL to 4.2 mL when 3% was added. At a high temperature and high pressure (HTHP), the minimum fluid loss was 14.4 mL with the addition of 2~3% polymer microspheres. As a result, polymer microspheres used as a plugging agent effectively reduced the API and HTHP filtration of oil-based drilling fluids and showed good thermal stability under high-temperature and high-pressure conditions.

### 4.2. Evaluation of Plugging Performance of Polymer Microspheres

**Figure 14:**
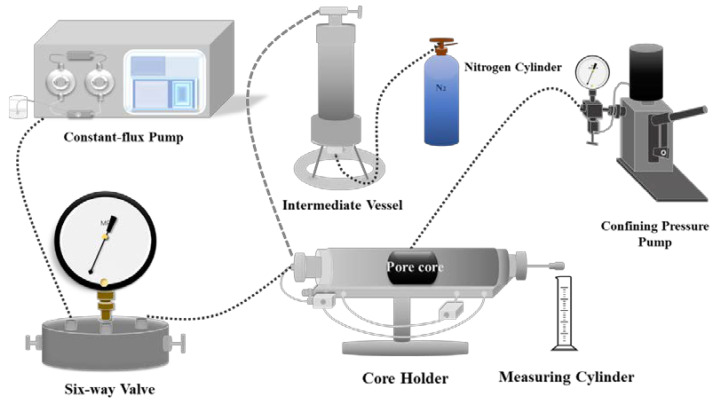
Liquid measuring device (pore core is placed in core gripper).

A 2.5 cm × 2.5 cm core was made according to the ratio of cement, sand, and water at 1,5,1. A liquid measuring device (as shown in Figure 14) was used to measure its permeability before and after the injection of oil-based drilling fluid, and then the sealing effect of polymer microspheres with different concentrations on pore cores was judged according to the difference between the two fields.

**Figure 15:**
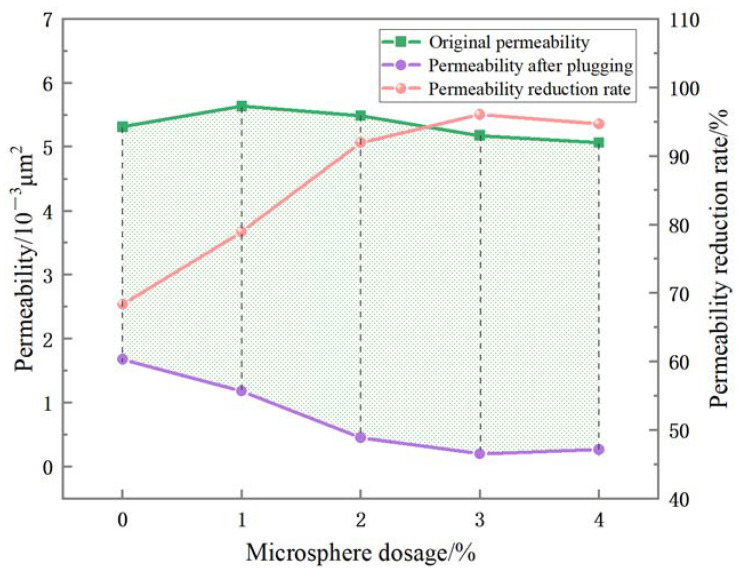
Effect of different concentrations of the polymer microspheres added to oil-based drilling fluid on the permeability and reduction rate of permeability of pore cores.

Polymer microspheres of different concentrations were added to the oil-based drilling fluid for aging, and then put into the liquid measuring device for an experiment to be conducted. The experimental results depicted in Figure 15 clearly demonstrate that the permeability of the oil-based drilling fluid more significantly decreases after flooding into the pore core and subsequent water flooding, as compared to that during the initial water flooding stage. The green area in the figure also shows the change in the difference before and after. When the polymer microspheres content reached 3%, the permeability difference reached the maximum, and the permeability reduction rate reached 96.04% (as shown in Table 1). It can be concluded that the optimal amount of polymer microspheres in oil-based drilling fluid is 3%.

## 5. Conclusions

Polymer microspheres were synthesized, with acrylamide, 2-acrylamide-2-methylpropane sulfonic acid, and acrylic acid acting as main monomers, via inverse emulsion polymerization. The polymer microspheres had good compatibility with oil-based drilling fluid. Infrared spectroscopy, scanning electron microscopy, and thermogravimetric analysis were used to characterize the polymer microspheres. The results showed that the synthesized polymer microspheres had uniform particle sizes, with an average size of 300~600 nm, were spherical, and contained corresponding functional groups. The properties of polymer microspheres were assessed by measuring various parameters after adding different concentrations of polymer microspheres to the oil-based drilling fluid. The apparent viscosity and plastic viscosity of the oil-based drilling fluid measured in the experiment show that polymer microspheres have a little influence on the rheological properties of oil-based drilling fluid. The measured emulsion-breaking voltage of the oil-based drilling fluid also reflects that the polymer microspheres have no adverse effect on the emulsion stability of the oil-based drilling fluid. When the polymer microspheres with 3% concentration were added to the oil-based drilling fluid, the filtration loss decreased from 6.8 mL to 4.2 mL in the experiment at a normal temperature and pressure, which decreased by 38.24%. In the high-temperature and high-pressure experiment, the filtration loss was reduced from 22.4 mL to 14.4 mL, which was reduced by 35.71%. Subsequently, the oil-based drilling fluid with different concentrations of polymer microspheres was used for the liquid measurement test, and the permeability and permeability reduction rates were compared before and after the experiment. The results show that the permeability reduction rate of oil-based drilling fluid reached 96.04% after the addition of 3% polymer microspheres, and the effect was the best. As a plugging agent, polymer microspheres are well adapted to oil-based drilling fluids. When the oil-based drilling fluid enters the formation, microspheres undergo irregular deformation and are extruded into the microporous fractures. At the same time, they can absorb water and expand in the leakage channel to form a compact pressurized seal, thus achieving the plugging effect.

## Figures and Tables

**Table 1:** Variation parameters of permeability and permeability reduction rate of water flooding before and after adding oil-based drilling fluid.

Microsphere Addition/%	Before and After Water Flooding	Flowing Pressure/MPa	Liquid Volume/mL	Time/s	Permeability/10^−3^ μm^2^	/%
0	original	0.36	6	187	5.315	68.39
		0.36	8	248		
	after plugging	1.24	2	57	1.68	
		1.22	8	232		
1	original	0.32	2	66	5.635	78.97
		0.34	2	62		
	after plugging	1.54	8	261	1.185	
		1.52	2	66		
2	original	0.34	2	64	5.49	91.97
		0.34	4	127		
	after plugging	1.56	2	170	0.455	
		1.56	4	332		
3	original	0.36	2	64	5.18	96.04
		0.36	4	127		
	after plugging	1.34	2	427	0.205	
		1.34	4	871		
4	original	0.36	4	130	5.07	94.67
		0.36	6	196		
	after plugging	1.26	2	352	0.27	
		1.26	4	697		

## Data Availability

Not applicable.
